# One-hour postload glucose levels predict mortality from cardiovascular diseases and malignant neoplasms in healthy subjects

**DOI:** 10.1093/pnasnexus/pgaf179

**Published:** 2025-06-02

**Authors:** Daiki Sato, Junta Imai, Michihiro Satoh, Yohei Kawana, Hiroto Sugawara, Akira Endo, Masato Kohata, Junro Seike, Hiroshi Komamura, Toshihiro Sato, Shinichiro Hosaka, Yoichiro Asai, Shinjiro Kodama, Kei Takahashi, Keizo Kaneko, Yukako Tatsumi, Takahisa Murakami, Takuo Hirose, Azusa Hara, Ryusuke Inoue, Kei Asayama, Hirohito Metoki, Atsushi Hozawa, Masahiro Kikuya, Yutaka Imai, Takayoshi Ohkubo, Hideki Katagiri

**Affiliations:** Department of Diabetes, Metabolism and Endocrinology, Tohoku University Graduate School of Medicine, 2-1 Seiryo-machi, Aoba-ku, Sendai, Miyagi 980-8575, Japan; Department of Diabetes, Metabolism and Endocrinology, Tohoku University Graduate School of Medicine, 2-1 Seiryo-machi, Aoba-ku, Sendai, Miyagi 980-8575, Japan; Division of Public Health, Hygiene and Epidemiology, Faculty of Medicine, Tohoku Medical and Pharmaceutical University, Sendai, Miyagi 983-8536, Japan; Department of Preventive Medicine and Epidemiology, Tohoku Medical Megabank Organization, Tohoku University, Sendai, Miyagi 980-0872, Japan; Department of Diabetes, Metabolism and Endocrinology, Tohoku University Graduate School of Medicine, 2-1 Seiryo-machi, Aoba-ku, Sendai, Miyagi 980-8575, Japan; SiRIUS Institute of Medical Research, Tohoku University, Sendai, Miyagi 980-0872, Japan; Department of Diabetes, Metabolism and Endocrinology, Tohoku University Graduate School of Medicine, 2-1 Seiryo-machi, Aoba-ku, Sendai, Miyagi 980-8575, Japan; Department of Diabetes, Metabolism and Endocrinology, Tohoku University Graduate School of Medicine, 2-1 Seiryo-machi, Aoba-ku, Sendai, Miyagi 980-8575, Japan; Department of Diabetes, Metabolism and Endocrinology, Tohoku University Graduate School of Medicine, 2-1 Seiryo-machi, Aoba-ku, Sendai, Miyagi 980-8575, Japan; Department of Diabetes, Metabolism and Endocrinology, Tohoku University Graduate School of Medicine, 2-1 Seiryo-machi, Aoba-ku, Sendai, Miyagi 980-8575, Japan; Department of Diabetes, Metabolism and Endocrinology, Tohoku University Graduate School of Medicine, 2-1 Seiryo-machi, Aoba-ku, Sendai, Miyagi 980-8575, Japan; Department of Diabetes, Metabolism and Endocrinology, Tohoku University Graduate School of Medicine, 2-1 Seiryo-machi, Aoba-ku, Sendai, Miyagi 980-8575, Japan; Department of Diabetes, Metabolism and Endocrinology, Tohoku University Graduate School of Medicine, 2-1 Seiryo-machi, Aoba-ku, Sendai, Miyagi 980-8575, Japan; Department of Diabetes, Metabolism and Endocrinology, Tohoku University Graduate School of Medicine, 2-1 Seiryo-machi, Aoba-ku, Sendai, Miyagi 980-8575, Japan; Department of Diabetes, Metabolism and Endocrinology, Tohoku University Graduate School of Medicine, 2-1 Seiryo-machi, Aoba-ku, Sendai, Miyagi 980-8575, Japan; Department of Diabetes, Metabolism and Endocrinology, Tohoku University Graduate School of Medicine, 2-1 Seiryo-machi, Aoba-ku, Sendai, Miyagi 980-8575, Japan; Department of Diabetes, Metabolism and Endocrinology, Tohoku University Graduate School of Medicine, 2-1 Seiryo-machi, Aoba-ku, Sendai, Miyagi 980-8575, Japan; Department of Hygiene and Public Health, Teikyo University School of Medicine, Tokyo 173-8605, Japan; Division of Public Health, Hygiene and Epidemiology, Faculty of Medicine, Tohoku Medical and Pharmaceutical University, Sendai, Miyagi 983-8536, Japan; Department of Preventive Medicine and Epidemiology, Tohoku Medical Megabank Organization, Tohoku University, Sendai, Miyagi 980-0872, Japan; Division of Aging and Geriatric Dentistry, Department of Rehabilitation Dentistry, Tohoku University Graduate School of Dentistry, Sendai, Miyagi 980-8575, Japan; Division of Integrative Renal Replacement Therapy, Faculty of Medicine, Tohoku Medical and Pharmaceutical University, Sendai, Miyagi 983-8536, Japan; Laboratory of Social Pharmacy and Epidemiology, Showa Pharmaceutical University, Machida, Tokyo 194-8543, Japan; Department of Medical Information Technology Center, Tohoku University Hospital, Sendai, Miyagi 980-8574, Japan; Department of Hygiene and Public Health, Teikyo University School of Medicine, Tokyo 173-8605, Japan; Tohoku Institute for Management of Blood Pressure, Hanamaki, Iwate 028-3203, Japan; Division of Public Health, Hygiene and Epidemiology, Faculty of Medicine, Tohoku Medical and Pharmaceutical University, Sendai, Miyagi 983-8536, Japan; Department of Preventive Medicine and Epidemiology, Tohoku Medical Megabank Organization, Tohoku University, Sendai, Miyagi 980-0872, Japan; Tohoku Institute for Management of Blood Pressure, Hanamaki, Iwate 028-3203, Japan; Department of Preventive Medicine and Epidemiology, Tohoku Medical Megabank Organization, Tohoku University, Sendai, Miyagi 980-0872, Japan; Department of Preventive Medicine and Epidemiology, Tohoku Medical Megabank Organization, Tohoku University, Sendai, Miyagi 980-0872, Japan; Department of Hygiene and Public Health, Teikyo University School of Medicine, Tokyo 173-8605, Japan; Tohoku Institute for Management of Blood Pressure, Hanamaki, Iwate 028-3203, Japan; Department of Hygiene and Public Health, Teikyo University School of Medicine, Tokyo 173-8605, Japan; Tohoku Institute for Management of Blood Pressure, Hanamaki, Iwate 028-3203, Japan; Department of Diabetes, Metabolism and Endocrinology, Tohoku University Graduate School of Medicine, 2-1 Seiryo-machi, Aoba-ku, Sendai, Miyagi 980-8575, Japan; SiRIUS Institute of Medical Research, Tohoku University, Sendai, Miyagi 980-0872, Japan

**Keywords:** cardiovascular diseases, malignant neoplasms, mortality, 1-h postload plasma glucose, oral glucose tolerance tests

## Abstract

Little is known about biological markers, at levels within their normal ranges which might predict future mortality. We aimed at identifying possible predictors of future death in participants before pathological conditions manifest. We analyzed data from a population-based prospective cohort study (the Ohasama study), comprised of 993 participants who underwent 75-g oral glucose tolerance tests (OGTTs). We collected blood parameters, including those measured during OGTTs, and divided the study population into two groups based on the median value of each parameter, followed by analyses of mortality during follow-up in both groups. In addition, we extracted subjects with normal glucose tolerance (NGT) (*n* = 595) and analyzed the association between 1-h postload plasma glucose during OGTTs (1-hrPG) and mortality as well as the causes of death. Among all parameters evaluated, 1-hrPG was found to be most significantly associated with all-cause mortality during the mean follow-up of 14.3 years. When we focused on subjects with NGT, Harrell's C concordance index analysis revealed a cut-off of 1-hrPG ≥170 mg/dL to be most strongly associated with all-cause mortality (0.8066). The Kaplan–Meier plots showed nearly double the proportion of the 1-hrPG ≥170 group to have died as compared with the 1-hrPG <170 group throughout the follow-up period after the third year. Cardiovascular diseases and malignant neoplasms both strongly contributed to the increased mortality in the high 1-hrPG group. Thus, 1-hrPG ≥170 is a powerful predictor of future death in subjects with NGT. Atherosclerotic and malignant diseases both contributed to the increased mortality.

**Significance statement**

Analysis of the population-based prospective Ohasama cohort database revealed, 1-h postload plasma glucose (1-hrPG) during oral glucose tolerance tests to be significantly associated with mortality during a mean follow-up period of 14.3 years. In addition, in subjects with normal glucose tolerance (NGT), a 1-hrPG of 170 mg/dL or more, was found to be a very strong predictor of all-cause mortality. Cancer and heart diseases strongly contributed to the increased mortality in the high 1-hrPG group. Thus, the 1-hrPG level is potentially useful for early detection and interventions aimed at cancer and heart diseases. Furthermore, focusing on 1-hrPG may be a new approach to diagnosing diabetes, because it takes mortality, the most important clinical outcome, into account.

## Introduction

Several parameters at pathological levels, i.e. outside their normal ranges, such as high blood pressure, high plasma glucose, and high body mass index (BMI), are known to be leading risk factors for mortality (1). However, little is known about biological predictors of future death prior to pathological conditions manifesting. If such predictors of future mortality in healthy subjects could be identified, and, furthermore, if the related causes of death could be ascertained, early detection of the associated life-threating conditions and interventions aimed at preventing fatal diseases might well become possible. This would potentially extend healthy life expectancy. Therefore, we herein attempted to identify such predictors of future mortality using a population-based cohort dataset.

The Ohasama study is a population-based prospective investigation that has been ongoing since 1986 in Iwate prefecture, Japan. Several important findings obtained from the Ohasama study (2–5) have been used to determine the diagnostic criteria for home hypertension in the World Health Organization-International Society of Hypertension (WHO/ISH) guidelines (6). Participant death and causes of death, such as cardiovascular diseases, cerebrovascular diseases and malignant neoplasms, as well as several blood parameters, including those from 75-g oral glucose tolerance tests (OGTTs), have also been continuously collected from the Ohasama cohort. Herein, employing the database of the Ohasama cohort, among all parameters evaluated in this study, 1-h postload plasma glucose (1-hrPG) during OGTTs was found to be most significantly associated with mortality.

Currently, diabetes is diagnosed by a combination of fasting plasma glucose levels, 2-h postload plasma glucose during OGTTs (2-hrPG), a random plasma glucose and HbA1c (7, 8). These diagnostic criteria were determined based mainly on the aim of preventing the development of diabetic microvascular complications (9–11). The 1-hrPG has, however, attracted interest as a predictor of developing diabetes. Previous studies have shown the usefulness of 1-hrPG for predicting future diabetes (12–16). In addition, several longitudinal studies have shown all-cause mortality to be predicted by 1-hrPG. A study conducted by the Chicago Heart Association Project in Industry suggested that, in middle-aged to elderly men and women, a 1-hrPG of 200 mg/dL or more is an independent risk factor for 22-year mortality from coronary heart disease and all-cause mortality (17). Similarly, based on the population-based Erfurt Male Cohort Study, a 1-hrPG of 200 mg/dL or more was reported to be a long-term predictor of all-cause mortality during 30-year period in middle-aged men (18).

Subsequently, two other studies found a 1-hrPG of 155 mg/dL or more to be associated with all-cause mortality in Caucasians (19, 20). In addition, a group from China recently showed that, when elderly male participants without diabetes were categorized into 1-hrPG tertiles, the highest tertile (mean 1-hrPG of 210.6 ± 21.6 mg/dL) had significantly elevated all-cause mortality as compared with the lowest tertile (mean 1-hrPG of 133.2 ± 18 mg/dL) during a 20-year follow-up period (21). In these studies, as only the specific values of 1-hrPG were analyzed, how strongly 1-hrPG levels are associated with mortality and what value of 1-hrPG would be most appropriate for predicting future death have yet to be elucidated. Furthermore, the causes of deaths related to high 1-hrPG remain unclear. Determining an appropriate cut-off and identifying the causes of death associated with high 1-hrPG might facilitate early detection and intervention, aimed at the underlying causes, to prevent mortality. Therefore, we herein endeavored to elucidate these important issues in subjects with normal glucose tolerance (NGT).

In the present study, by calculating the Harrell's C concordance index, a 1-hrPG of 170 mg/dL or more, was found to be a very strong predictor of all-cause mortality, with the highest significance, in subjects with NGT. Furthermore, we explored which causes of death contributed to the increased mortality observed in the NGT group with 1-hrPG ≥170 and revealed that increased incidences of cardiovascular death and death from malignant neoplasms were strongly associated with 1-hrPG ≥170 mg/dL. Thus, 1-hrPG ≥170 may be a useful cut-off value for early detection and interventions, targeting cardiovascular diseases and malignant neoplasms, with the aim of extending lifespan.

## Materials and methods

### Study population

This study constituted part of the Ohasama study, an ongoing community-based project which has been underway since 1986. Socioeconomic and demographic characteristics of this region and details of the study were previously described (22).

Participants were males and females without a history of diabetes mellitus, at least 35 years of age, who resided in Ohasama, a rural community in Iwate Prefecture, Japan. Participants who underwent OGTT at least once during the period from 1997 to 2013 were included (*n* = 1137), but participants were not eligible if they had received less than 4 years of follow-up (*n* = 133), had incomplete outcome data (*n* = 7), or their OGTT data results were incomplete (*n* = 4). Ultimately, we analyzed 993 participants who underwent OGTTs. The mean age and BMI of subjects were 63.1 years and 23.7 kg/m^2^, respectively, and 67.8% of the subjects were female (Supplementary Table S1). A mean follow-up period was 14.3 years and the incidence of all-cause mortality was 184. From the database of the Ohasama study, we extracted all hematological and biochemical blood parameters collected in the study, such as blood cell counts, lipids, renal and hepatic markers, uric acid, serum proteins, and electrolytes. In addition, OGTT parameters, such as blood levels of glucose and insulin at baseline, and at 1 h and at 2 h after glucose loading, were extracted. Then, we divided the study population into two groups, i.e. the high and low value groups, based on the median value of each parameter, and analyzed the incidence of mortality during follow-up in both groups.

The study was approved by the Institutional Review Board of Teikyo University, Tohoku University Graduate School of Medicine and Tohoku Medical and Pharmaceutical University. All participants gave informed consent.

### Outcomes

The National Vital statistics of the Ministry of Health, Labor, and Welfare, Japan, for determining causes of death were obtained from all cohort members. In accordance with the Family Registration Law in Japan, all death certificates are forwarded to the Ministry of Health, Labor and Welfare via the public health center in the area of residence. Death certificates were also used for the ascertainment and definition of fatal events if available. The last follow-up date was 2017 December 31.

For individuals who died during the follow-up period, the underlying cause of death was classified, according to the recommendations of the International Classification of Disease-10. We collected cardiovascular, cerebrovascular, and malignant neoplasm mortality data. The details of the adjudication of death causes used in the Ohasama study were previously described (3). We defined other-cause mortality as deaths other than those due to cardiovascular diseases, cerebrovascular diseases, and malignant neoplasms.

### Oral glucose tolerance tests

Using blood samples collected before the glucose load, i.e. in the fasting state, plasma glucose, and serum insulin and other blood parameters were measured. Using blood samples at both 60 and 120 min after oral administration of a 75-g glucose-equivalent carbohydrate (Trelan G; Ajinomoto Pharma, Tokyo, Japan), plasma glucose and serum insulin levels were measured. Glucose levels were measured enzymatically at SRL (Tokyo, Japan). Based on the results of OGTTs, we defined glycemic categories. NGT was defined according to the WHO criteria (23).

### Definition of covariates

Study nurses or physicians administered a standardized questionnaire that included questions concerning the medical history, and the smoking and alcohol consumption habits of each patient. Habitual smoking was defined as the current smoker. Drinking habit was defined as the participants currently drinking alcohol. Home blood pressures and pulse rates were measured as previously reported (24).

Glucose and fibrinogen in plasma, insulin, total cholesterol, triglyceride, lipoprotein a, creatinine, uric acid, sodium and potassium in serum, and HbA1c and hemoglobin in blood were measured enzymatically. Because HbA1c has used herein was the Japan Diabetes Society measurement values (JDS), we converted these values to the National Glycohemoglobin Standardization Program measurement values (NGSP), employing the following formula: HbA1c (NGSP (%)) = 1.02 × HbA1c (JDS (%)) + 0.25 (25).

As an index of insulin resistance, homeostasis model assessment of insulin resistance (HOMA-IR) was calculated with the following formula: Fasting insulin (μU/mL) × Fasting blood glucose (mg/dL)/405 (26).

### Statistical analyses

Fasting, 1- and 2-h postload glucose and insulin, blood pressure, and other blood parameters were used to divide the subjects into two groups based on the 50th percentile line. The baseline characteristics are expressed as means ± SD unless otherwise noted.

We used Student's t test to compare age, BMI, glucose, insulin, HbA1c, blood pressure, HOMA-IR, and other blood parameters unless otherwise indicated. We used the chi-squared test to compare sex, habitual smoking, alcohol drinking habit, and family history of diabetes. To construct the survival curve, Kaplan–Meier analysis with the log-rank test was used for all-cause mortality. We calculated hazard ratios (HRs) to compare the differences in all-cause mortality and deaths due to cardiovascular diseases and malignant neoplasms by using Cox proportional hazards models. HRs were adjusted for sex, BMI, age, family history of diabetes, triglycerides, the presence or absence of hypertension, and OGTT parameters other than 1-hrPG. To evaluate the optimal cut-off value for 1-hrPG when assessing all-cause mortality, we calculated the Harrell's C concordance index within the interquartile range of each 1-hrPG. Kaplan–Meier curves were plotted for the cumulative incidences of events, and the log-rank test compared group differences.

In all analyses, a *P*-value < 0.05 was considered to indicate a statistically significant difference. All data were statistically analyzed using SAS version 9.4 software (SAS Institute, Cary, NC, USA).

## Results

### High 1-hrPG during OGTT is associated with all-cause mortality in the entire subject population

From the database of the Ohasama study, we analyzed 993 participants who underwent OGTTs. First, we divided the study population into two groups, i.e. the high and low value groups, based on the median value of each parameter, and analyzed the incidence of mortality during follow-up in both groups (Table 1). Notably, among all parameters evaluated in the present study, 1-hrPG levels during OGTTs were most significantly associated with all-cause mortality. In contrast, other parameters associated with glucose metabolism and insulin action, including fasting and 2-hrPG, all insulin levels during OGTTs, HOMA-IR, and HbA1c, did not show statistical significance (Table 1).

**Table 1.** Adjusted HRs of each parameter for all-cause mortality.

Category	Hazard ratio (95% CI)	*P*-value
Fasting blood glucose	1.264 (0.875–1.824)	0.2117
1-h postload glucose	1.620 (1.119–2.347)	0.0107^a^
2-h postload glucose	1.194 (0.841–1.693)	0.3213
Fasting insulin	1.244 (0.855–1.809)	0.253
1-h postload insulin	1.076 (0.760–1.524)	0.6783
2-h postload insulin	1.412 (0.966–2.064)	0.0748
HOMA-IR	1.176 (0.811–1.705)	0.3918
HbA1c	1.211 (0.846–1.733)	0.2948
Total cholesterol	0.660 (0.461–0.944)	0.0227
Triglycerides	0.992 (0.694–1.418)	0.9659
Home systolic blood pressure	1.025 (0.714–1.471)	0.8924
Home diastolic blood pressure	0.961 (0.671–1.376)	0.8281
Fibrinogen	0.811 (0.576–1.142)	0.2307
Lipoprotein a	0.780 (0.553–1.101)	0.1578
Hemoglobin	0.678 (0.454–1.014)	0.0583
Creatinine	0.869 (0.593–1.274)	0.4725
Uric acid	0.955 (0.650–1.401)	0.8125
Sodium	0.773 (0.544–1.097)	0.1496
Potassium	0.877 (0.622–1.238)	0.4556

Adjusted HRs are estimated with the Cox proportional hazard model. All estimated HRs are adjusted for sex, BMI, age, family history of diabetes, triglycerides, and the presence or absence of obvious hypertension.

BMI, body mass index; HOMA-IR, homeostasis model assessment of insulin resistance.

^a^ *P* < 0.05 assessed by Student's t test.

The median value of 1-hrPG levels in our subjects was 162 mg/dL. The 993 participants were divided into two groups based on 1-hrPG values of 162 mg/dL or more (1-hrPG ≥162) and <162 mg/dL (1-hrPG <162). Then, mortalities of these two groups were compared during the follow-up periods, employing the Cox proportional hazard model. The analyses were performed after adjustment for factors including age, gender, BMI, family history of diabetes, triglycerides, and the presence or absence of hypertension, as well as habitual smoking. The factors for adjustment were selected according to previous reports (4, 20, 27). These analyses revealed 1-hrPG ≥162 to be significantly and strongly associated with all-cause mortality (HR = 1.620 [95% CI 1.119–2.347], *P* = 0.0107) (Table 1).

**Fig. 1.**
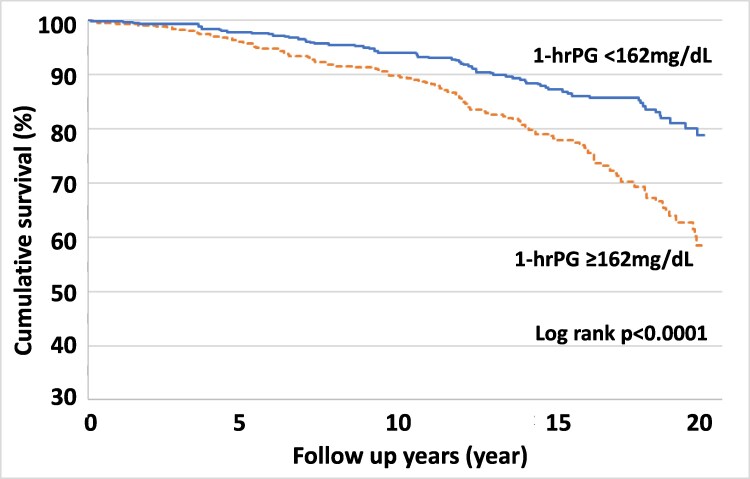
The Kaplan–Meier plot showing unadjusted survival all-cause deaths. The estimated cumulative all-cause survivals of the groups with 1-hrPG <162 mg/dL and 1-hrPG ≥162 mg/dL are shown. The solid line is a survival curve of the 1-hrPG <162 mg/dL group, and the dashed line the survival curve of the 1-hrPG ≥162 mg/dL group. 1-hrPG, 1-h postload plasma glucose during OGTTs.

Next, we explored cumulative survival in both groups during a mean follow-up period of 14.3 years. The Kaplan–Meier plot consistently showed that cumulative survival declines were significantly steeper in the high than in the low 1-hrPG group (log rank *P* < 0.0001) (Fig. 1). Throughout the follow-up period, almost double the proportion of the high 1-h group died as compared with the low 1-hrPG group. Strikingly, at the follow-up time-point of 20.4 years, more than 40% of the high 1-hrPG group had died, while the death rate was ∼20% in the low 1-hrPG group. These results indicate that a high 1-hrPG level strongly predicts all-cause mortality in the entire subject population of the present study.

### One-hour PG level of 170 mg/dL or more is a strong predictor of all-cause mortality in NGT subjects

Subjects with diabetes tend not to undergo OGTTs. In addition, as described in the introduction, we aimed to identify biological markers which might serve as predictors of future mortality in subjects without apparent diseases. Therefore, to examine the capacity of 1-hrPG to predict future mortality of such subjects, we extracted those shown to have NGT based on the OGTT results according to the WHO criteria (23), and analyzed the association between their 1-hrPG levels and all-cause mortality.

The number of NGT subjects who underwent OGTTs was 595. Baseline characteristics of these subjects are shown in Supplementary Table S2. Their mean age and BMI were 62.0 years and 23.2 kg/m^2^, respectively, and 68.2% of the subjects were female (Supplementary Table S2). A mean follow-up period was 14.3 years and incidence of all-cause mortality was 108. Notably, not only in the entire subject population but also in the NGT subjects, 1-hrPG ≥162 mg/dL was significantly associated with all-cause mortality after adjustment for the aforementioned factors (HR = 1.642 [95% CI 1.024–2.634], *P* = 0.0396). Thus, 1-hrPG levels are associated with the future mortality in NGT subjects as well.

We next attempted to determine the 1-hrPG cut-off value with the highest significance for predicting mortality. To this end, we examined the associations between all-cause mortality and 1-hrPG values ranging from 114 mg/dL to 175 mg/dL, essentially the interquartile range of the 1-hrPG values in NGT subjects, and determined which 1-hrPG level is most significantly associated with all-cause mortality. These analyses revealed the Harrell's C concordance index at 170 mg/dL to be the highest, 0.8066, among all of the 1-hrPG values analyzed (Supplementary Fig. S1).

**Fig. 2.**
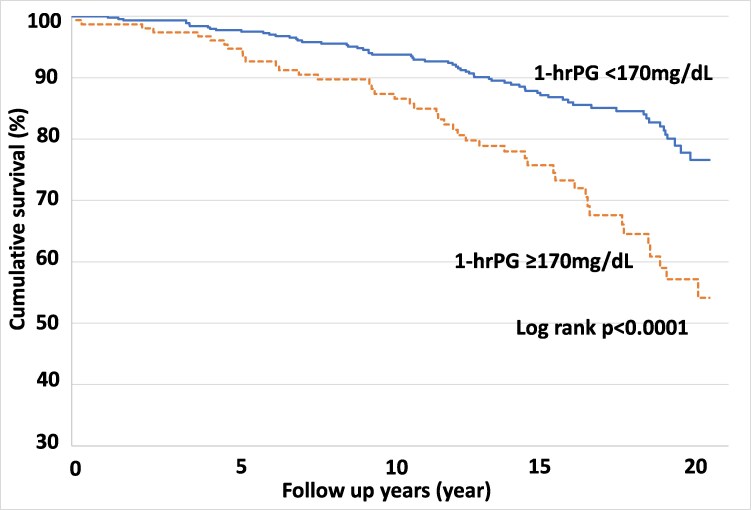
The Kaplan–Meier plot showing unadjusted survival from all-cause deaths in NGT subjects. The estimated cumulative survivals of the groups with 1-hrPG <170 mg/dL and 1-hrPG ≥170 mg/dL are shown. The solid line is the survival curve of the 1-hrPG <170 mg/dL group, and the dashed line is the survival curve of the 1-hrPG ≥170 mg/dL group. NGT, normal glucose tolerance; 1-hrPG, 1-h postload plasma glucose levels during OGTTs.

Then, we analyzed cumulative survival in the group with 1-hrPG level of 170 mg/dL or more (1-hrPG ≥170) as compared to those with values below 170 mg/dL (1-hrPG <170) during the follow-up periods. The Kaplan–Meier plots showed that, after the third year of follow-up, nearly double the proportion of the 1-hrPG ≥170 group had died as compared with the 1-hrPG <170 group, and this remained true throughout the remainder of the follow-up period (Log rank *P* < 0.0001) (Fig. 2). In addition, 1-hrPG ≥170 was significantly associated with all-cause mortality in the NGT subjects after adjustment for the aforementioned factors (HR = 1.821 [95% CI 1.128–2.941], *P* = 0.0142). These results indicate that 170 mg/dL is the most significant cut-off 1-hrPG value for predicting future mortality in NGT subjects.

### One-hrPG ≥170 is associated with increased incidence of cardiovascular death and death from malignant neoplasms in NGT subjects

**Fig. 3.**
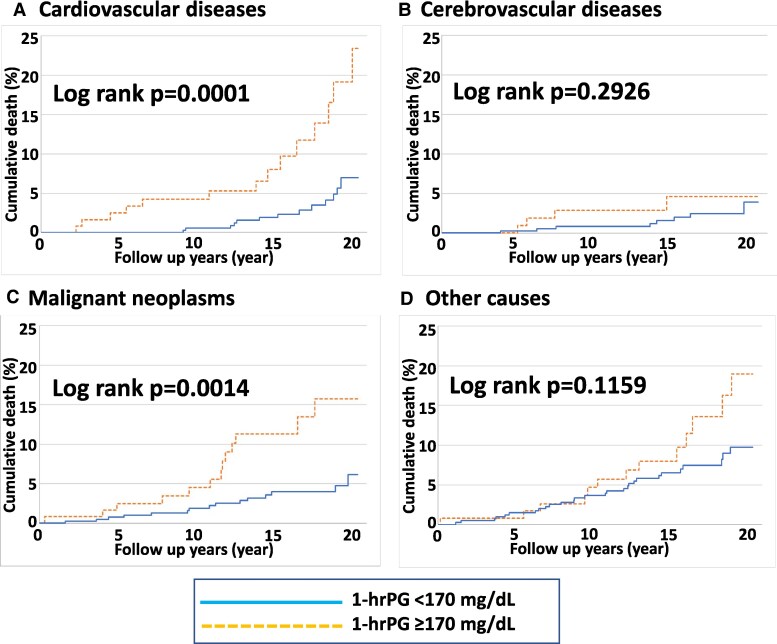
The Kaplan–Meier plot showing cumulative death from each cause in NGT subjects. The estimated cumulative death rate of the groups with 1-hrPG <170 mg/dL and 1-hrPG ≥170 mg/dL are shown. The solid line is the cumulative death curve of the 1-hrPG <170 mg/dL group, and the dashed line is the cumulative death curve of the 1-hrPG ≥170 mg/dL group. A) Cumulative rate of death due to cardiovascular disease. B) Cumulative rate of death due to cerebrovascular disease. C) Cumulative rate of death due to malignant neoplasms. D) Cumulative rate of death due to other causes. NGT, normal glucose tolerance; 1-hrPG, 1-h postload plasma glucose levels during OGTTs.

Finally, we explored which causes of death contributed to the increased mortality in the NGT group with 1-hrPG ≥170. Incidence numbers for all-cause mortality, deaths due to cardiovascular diseases, cerebrovascular diseases, malignant neoplasms, and other-cause mortality were 108, 27, 12, 28, and 41, respectively. Incidences of death due to cardiovascular diseases (Fig. 3A) and malignant neoplasms (Fig. 3C), were significantly higher in the 1-hrPG ≥170 NGT group, whereas incidences of cerebrovascular death (Fig. 3B) and other-cause mortality (Fig. 3D) did not differ significantly between the 1-hr PG ≥170 NGT and 1-hr PG <170 NGT groups. Notably, as observed in the analysis of all-cause mortality (Fig. 2), ratios of mortality caused by cardiovascular diseases differed between the two groups at early periods, less than and around 5 years, after undergoing OGTTs, while malignant neoplasm was observed to strongly contribute to mortality at the latter follow-up period. In addition, after adjustment for the aforementioned factors, 1-hrPG ≥170 was significantly and strongly associated with deaths by both cardiovascular diseases (HR = 3.370 [95% CI 1.204–9.435], *P* = 0.0207) and malignant neoplasms (HR = 2.512 [95% CI 1.041–6.058], *P* = 0.0404). These results suggest that 1-hrPG ≥170 is a strong predictor of deaths caused by cardiovascular diseases and malignant neoplasms in the NGT population. These death causes both contribute markedly to high mortality observed in subjects with high 1-hrPG.

## Discussion

The first important finding in the present cohort study is that 1-hrPG levels during OGTTs are strongly associated with future mortality. When dividing the entire participants, who had undergone OGTTs, into two groups based on the median value of each parameter, we found 1-hrPG levels during OGTTs to be the most significantly associated with all-cause mortality among all parameters, including blood pressure levels which have well-known links to mortality, evaluated in the present study. Since our aim was to identify strong predictors of future death prior to the manifestation of pathological conditions, we next focused on subjects who had “normal” glucose tolerance based on the WHO criteria for OGTT results. In the NGT population as well, 1-hrPG ≥170 was identified as a strong predictor of future mortality during a mean follow-up period of 14.3 years.

Several reports have indicated that OGTT values are associated with mortality. When comparing participants with diabetes or impaired glucose tolerance (IGT) to those with NGT, diabetes/IGT was significantly associated with mortality (28, 29). Fasting plasma glucose and 2-hrPG levels during OGTTs were used for diagnosis of diabetes/IGT but 2-hrPG was reported to be a better predictor of all-cause mortality in diabetes/IGT subjects than fasting plasma glucose (7, 8). In the present study, however, 1-hrPG was shown to be a significantly better predictor of mortality than 2-hrPG in NGT subjects.

With regard to 1-hrPG, a 1-hrPG of 155 mg/dL or more was previously reported to be associated with all-cause mortality in Caucasians (19, 20), indicating that the association between 1-hrPG levels and future mortality is present across ethnic groups. These previous studies were aimed mainly at comparing the importance between 1-hrPG and 2-hrPG (or IGT), as indicators of future diabetes development, its complications and mortality. Only one cut-off value, 155 mg/dL for 1-hrPG, was used, because this value is reportedly predictive of IGT and subsequent type 2 diabetes development (30). Therefore, how strongly 1-hrPG levels contribute to mortality and whether the 155 mg/dL value of 1-hrPG is an appropriate cut-off as a predictor of future death have yet to be fully clarified. Another recent study showed a high 1-hrPG, i.e. 210.6 mg/dL, to be a long-term predictor of all-cause mortality in Chinese men of advanced age without diabetes (21). However, 1-hrPG of 210.6 mg/dL appears to be too high to categorize as normal. On the other hand, in the present study, applying Harrell's C concordance index analysis allowed a 1-hrPG cut-off value of 170 mg/dL to be identified as predicting future mortality, with this value yielding the highest statistical significance. Using this value as a cut-off, the HR of all-cause mortality was 1.821, which is similar to the reported HR based on the presence of diabetes (1.8) (31), with significance (*P* = 0.0142). In addition, the ages and observation periods were younger (63.1 years) and shorter (14.3 years) in the present study than those in the previous report of the Chinese men (74.0 years and 20 years, respectively). Thus, 1-hrPG ≥170 mg/dL is a more sensitive and significant marker allowing the prediction of future death in NGT subjects.

Most importantly, the present study revealed the causes of death, especially malignant neoplasms, associated with high 1-hrPG. There was no information about the related causes of deaths in the aforementioned previous reports. On the other hand, the present study showed cardiovascular diseases (*P* = 0.0207) and malignant neoplasms (*P* = 0.0404) to be highly significant as cause of death in NGT subjects with 1-hrPG ≥170, while incidences of cerebrovascular death and other-cause mortality did not differ significantly between the 1-hrPG ≥170 and 1-hrPG <170 groups. Strikingly, the HRs of cardiovascular death and death from malignant neoplasms, calculated after adjustments for the aforementioned factors, were very high, 3.370 and 2.512, respectively. Reported HRs of habitual smoking for deaths from ischemic heart diseases, pancreatic cancer, and gastric cancer are 3.0, 1.9, and 1.7, respectively (32). Although it is generally difficult to compare results among various populations, these findings suggest that 1-hrPG ≥170 has a major impact on mortality due to cardiovascular diseases and malignant neoplasms, especially in elderly subjects with NGT.

Although incidence numbers for cerebrovascular death were small, similar incidences of other-cause mortality between 1-hrPG ≥170 and 1-hrPG <170 groups indicate that death from atherosclerotic diseases and malignant neoplasms led to increases in overall mortality in the high 1-hrPG NGT group. Notably, three years after undergoing OGTTs, the death rate in the 1-hrPG ≥170 group was significantly higher than that in the 1-hrPG <170 group. Similarly, ratios of mortality caused by cardiovascular diseases differed between the two groups at early periods after undergoing OGTTs. Considering that we focused on NGT participants, significant differences observed after such short observation periods makes it unlikely that developing diabetes and its subsequent complications would mainly be responsible for the increased mortality. Alternatively, these findings suggest another common mechanism(s) underlying 1-hrPG elevation as well as cardiovascular diseases and malignant neoplasms.

It is well known that glucose elevation in postprandial states, called a glucose spike, leads to atherosclerosis development (33). However, intake of glucose, a terminally digested substrate, by drinking at OGTTs, readily raises blood glucose levels, while blood glucose elevation after dietary intake is much more modest. Considering that the relatively low 1-hrPG level, i.e. 170 mg/dL, is the optimal cut-off, it is unlikely that postmeal glucose spikes are the main cause of the mortality.

Insulin resistance in the periphery and the resultant hyperinsulinemia and chronic inflammation also reportedly promote the development of both atherosclerosis (34, 35) and neoplasms (36, 37). However, neither the serum insulin levels nor the HOMA-IR value was significantly associated with mortality, whereas 1-hrPG levels alone were found to be strongly associated with future death (Table 1). Therefore, insulin resistance is also unlikely to be the major mechanism underlying increased mortality in high 1-hrPG subjects.

In addition to increased peripheral insulin resistance, hepatic disposal of glucose affects blood glucose levels, especially after glucose loading. Disposal of absorbed glucose first occurs in the liver, and any glucose not undergoing hepatic disposal enters the peripheral circulation where we measure glucose levels. Thus, hepatic glucose disposal rates may be important for determining postload glucose levels, especially at early time-points after loading. In addition to glucose, many harmful substrates, such as carcinogens, derived from foods ingested daily, are also processed in the liver (38, 39). Therefore, we speculate that hepatic disposal capacity plays an important role in not only the suppression of postload glucose levels but also the removal of food-derived toxic substances, which may promote the development of atherosclerosis and/or malignant neoplasms when increased in the peripheral circulation. For instance, accumulation of slight but daily decreases in hepatic removal of these substances may contribute to death from malignancies at the latter follow-up period, i.e. after 10 years or more. In turn, 1-hrPG levels may serve as a surrogate marker of hepatic rates of disposal of these substances. This newly proposed mechanism may underscore the necessity for extensive future studies.

This study has several limitations. First, we did not follow the participants to determine whether or not they developed diabetes. However, as mentioned above, since increases in mortality manifested in the higher 1-hrPG groups after early periods after undergoing OGTTs, development of diabetes is unlikely to be the main mechanism of the increased mortality. Second, the number of laboratory items examined in this cohort was limited. However, our check-ups included several items which have already been established as being associated with mortality, such as BMI, the presence or absence of hypertension and habitual smoking. After adjustment for these factors, 1-hrPG levels remained strongly associated with future mortality. Third, our study was conducted on a single cohort. Notably, since the mean age of participants was 62.0 years, whether the results obtained from this cohort are applicable to younger populations also remains unclear.

In conclusion, we clarified that 1-hrPG ≥170 is a powerful predictor of future death in elderly subjects with NGT. Increased incidences of cardiovascular diseases and malignant neoplasms contribute to the high mortality of the NGT population. Thus, 1-hrPG levels should be regarded as a marker indicating a high risk of future death, and this marker may be useful for early detection and intervention for cardiovascular diseases and malignant neoplasms, thereby preventing mortality. Unraveling the mechanism(s) underlying the association between 1-hrPG and future mortality may lead to developing strategies for the prevention of lethal diseases and prolonging healthy life expectancy. Currently, the diagnostic criteria for diabetes (23) are defined based on the aim of preventing the development of diabetic microvascular complications and do not stress 1-hrPG levels. A new approach, focusing on 1-hrPG, may be very useful, because it takes mortality, the most important clinical outcome, into account.

## Supplementary Material

pgaf179_Supplementary_Data

## Data Availability

All data are included in the manuscript and/or supporting information.
