# Supplementary material for: One-hour postload glucose levels predict mortality from cardiovascular diseases and malignant neoplasms in healthy subjects
Source: PNAS Nexus. 2025 Jun 2;4(6):pgaf179. doi: 10.1093/pnasnexus/pgaf179 (PMC12163372; doi:10.1093/pnasnexus/pgaf179)
Supplement: pgaf179_Supplementary_Data [file pgaf179_supplementary_data.pdf]

Sato et al. Supplementary Table 1

Category	
Study population	993
Age, years	63.1 ± 9.4
Female (%)	67.8
BMI, kg/m^2	23.7 ± 3.2
Family history of diabetes, %	30.1
Smoking habitat, %	14.5
Drinking habitat, %	44.4
Home systolic blood pressure, mmHg	127.4 ± 15.6
Home diastolic blood pressure, mmHg	76.7 ± 9.3
Home pulse rate, b.p.m	64.6 ± 7.7
Fasting blood glucose, mg/dL	96.4 ± 11.8
1-h blood glucose, mg/dL	167.1 ± 53.8
2-h blood glucose, mg/dL	132.5 ± 45.3
Fasting insulin, μU/mL	5.9 ± 6.3
1-h insulin, μU/mL	54.3 ± 39.7
2-h insulin, μU/mL	46.2 ± 36.5
HOMA-IR	1.46 ± 2.31
HbA1c, %	5.6 ± 0.4
Total cholesterol, mg/dL	207.0 ± 33.2
Triglycerides, mg/dL	105.2 ± 61.5
Fibrinogen, mg/dL	292.0 ± 100.5
Lipoprotein a, mg/dL	22.1 ± 23.1
Hemoglobin, g/dL	13.5 ± 1.4
Creatinine, mg/dL	0.7 ± 0.2
Uric acid, mg/dL	4.7 ± 1.3
Sodium, mEq/L	141.9 ± 1.8
Potassium, mEq/L	4.4 ± 0.5

Supplementary Table 1: Baseline characteristics of the study population.
Data are presented as means ± SD of continuous parameters.
BMI, body mass index; HOMA-IR, homeostasis model assessment of insulin resistance.

Sato et al. Supplementary Table 2

Category	
Study population	595
Age, years	62.0 ± 10.0
Female (%)	68.2
BMI, kg/m^2	23.2 ± 3.1
Family history of diabetes, %	30.4
Smoking habitat, %	16.9
Drinking habitat, %	47.1
Home systolic blood pressure, mmHg	125.2± 15.4
Home diastolic blood pressure, mmHg	76.3 ± 9.5
Home pulse rate, b.p.m	64.7 ± 7.7
Fasting blood glucose, mg/dL	92.4 ± 7.0
1-h blood glucose, mg/dL	144.2 ± 42.7
2-h blood glucose, mg/dL	106.7 ± 20.8
Fasting insulin, μU/mL	5.1 ± 3.7
1-h insulin, μU/mL	54.4 ± 37.5
2-h insulin, μU/mL	35.6 ± 27.2
HOMA-IR	1.18 ± 0.88
HbA1c, %	5.5 ± 0.3
Total cholesterol, mg/dL	205.4 ± 32.6
Triglycerides, mg/dL	97.7 ± 54.6
Fibrinogen, mg/dL	292.0 ± 100.5
Lipoprotein a, mg/dL	24.0 ± 24.7
Hemoglobin, g/dL	13.4 ± 1.4
Creatinine, mg/dL	0.7 ± 0.2
Uric acid, mg/dL	4.6 ± 1.3
Sodium, mEq/L	141.9 ± 1.8
Potassium, mEq/L	4.4 ± 0.5

Supplementary Table 2: Baseline characteristics of NGT subjects.
Data are presented as means ± SD for the continuous parameters.
NGT, normal glucose tolerance; BMI, body mass index
HOMA-IR, homeostasis model assessment of insulin resistance.

Sato et al. Supplementary Figure 1

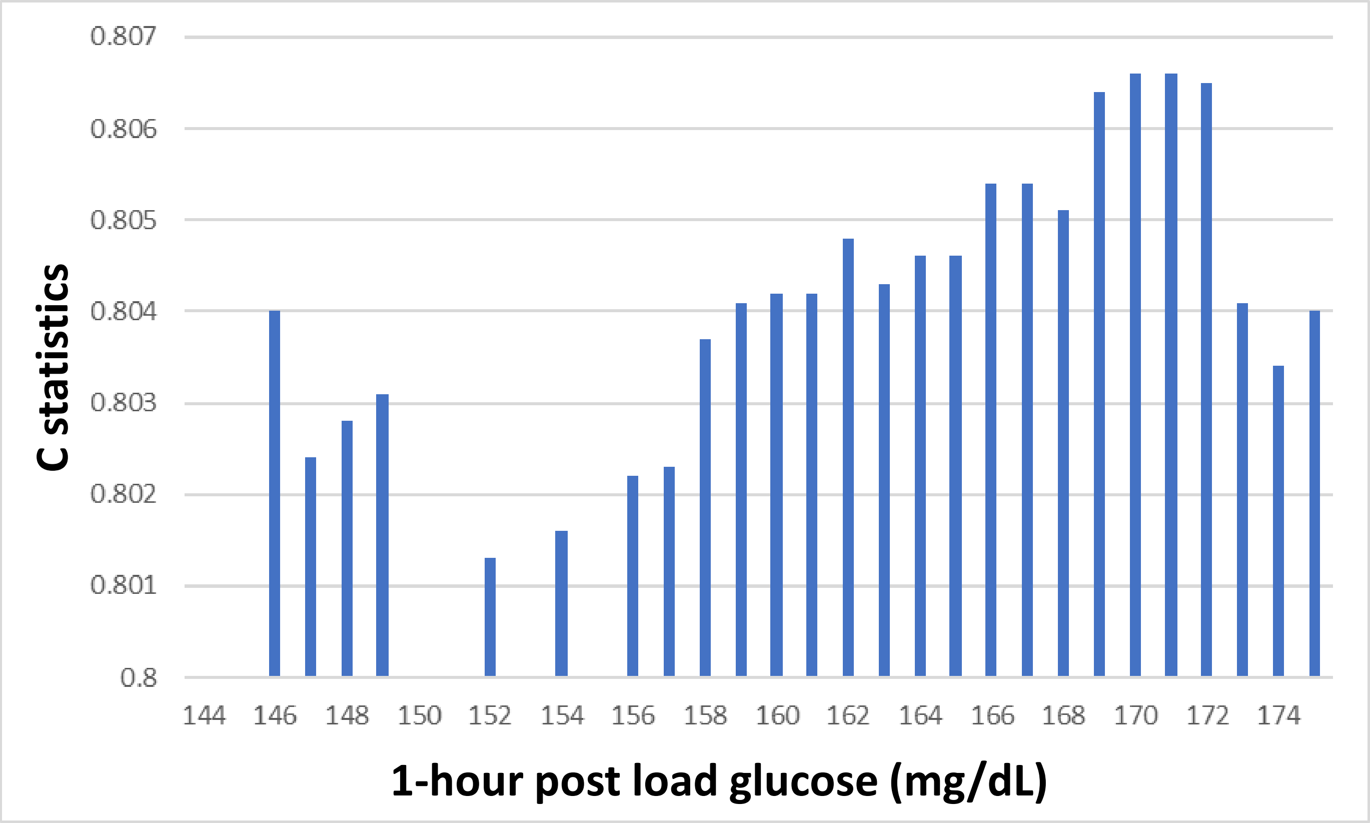

Supplementary Figure 1: Harrell's C statistic indexes stratified with 1-hrPG
Harrell's C statistics based on the Cox proportional hazard model.
Harrell's C concordance indexes are calculated with each 1-hrPG value as the cut-off.
No bars means that there are no significant association between 1-hrPG and all-cause mortality at these cut-off values.
1-hrPG: 1-hour post load plasma glucose levels during OGTTs.
